# Supplementary material for: Inpatient Outcomes of Patients Undergoing Robot-Assisted versus Laparoscopic Radical Cystectomy for Bladder Cancer: A National Inpatient Sample Database Study
Source: J Clin Med. 2024 Jan 29;13(3):772. doi: 10.3390/jcm13030772 (PMC10856412; doi:10.3390/jcm13030772)
Supplement: Supplementary file 1 [file jcm-13-00772-s001.zip › jcm-2739277-supplementary.pdf]

Journal of
Clinical Medicine

MDPI

**Supplementary Table S1.** The relationship between different operations and outcomes.

| Characteristic | In-hospital mortality | | Prolonged LOS [a, b] | |
|---|---|---|---|---|
| | Univariate | Multivariate | Univariate | Multivariate |
| | OR (95% CI) | aOR (95% CI) | OR (95% CI) | aOR (95% CI) |
| **Operation** | | | | |
| Laparoscopic | ref | ref | ref | ref |
| Robot-assisted | **0.43 (0.23, 0.78)** | **0.50 (0.28, 0.90)** | **0.68 (0.53, 0.86)** | **0.63 (0.49, 0.80)** |
| **Age** | | | | |
| 50-59 | ref | ref | ref | |
| 60-69 | 0.93 (0.33, 2.60) | 0.91 (0.28, 2.99) | 0.91 (0.72, 1.14) | 0.85 (0.67, 1.07) |
| 70-79 | 1.75 (0.67, 4.57) | 1.40 (0.45, 4.30) | 1.16 (0.92, 1.46) | 0.99 (0.76, 1.28) |
| 80+ | **3.29 (1.19, 9.09)** | 2.44 (0.73, 8.14) | **1.55 (1.20, 2.01)** | 1.27 (0.95, 1.71) |
| **Gender** | | | | |
| Male | 1.56 (0.76, 3.20) | | 0.99 (0.82, 1.18) | |
| Female | ref | | ref | |
| **Race** | | | | |
| White | ref | ref | ref | ref |
| Black | **2.50 (1.14, 5.49)** | **2.33 (1.05, 5.13)** | **1.40 (1.01, 1.95)** | |
| Hispanic | **2.68 (1.07, 6.72)** | 2.57 (0.97, 6.86) | 1.14 (0.77, 1.70) | |
| Others | 1.38 (0.43, 4.42) | 1.62 (0.50, 5.23) | 0.87 (0.60, 1.25) | |
| **Household income** | | | | |
| Quartile1 | 1.55 (0.73, 3.26) | | 0.86 (0.69, 1.08) | |
| Quartile2 | 1.26 (0.59, 2.70) | | 0.97 (0.80, 1.17) | |
| Quartile3 | 1.36 (0.68, 2.74) | | 0.90 (0.73, 1.11) | |
| Quartile4 | ref | | ref | |
| **Insurance status** | | | | |
| Medicare/Medicaid | ref | | ref | ref |
| Private including HMO | 0.58 (0.29, 1.16) | | **0.79 (0.67, 0.93)** | 0.93 (0.76, 1.12) |
| Self-pay/no-charge/other | 2.32 (0.81, 6.62) | | 0.66 (0.42, 1.02) | 0.70 (0.44, 1.11) |
| **CCI** | | | | |
| 0-1 | NA | NA | NA | NA |
| 2-3 | ref | ref | ref | ref |
| 4-5 | **3.35 (1.87, 6.00)** | **2.64 (1.43, 4.88)** | **1.61 (1.35, 1.92)** | **1.59 (1.32, 1.90)** |
| 6+ | **6.78 (3.53, 13.02)** | **5.61 (2.85, 11.06)** | **1.86 (1.43, 2.43)** | **1.89 (1.44, 2.48)** |
| **Hospital bed size** | | | | |
| Large | ref | | ref | |
| Medium | 1.34 (0.77, 2.34) | | 0.98 (0.80, 1.18) | |
| Small | 0.81 (0.35, 1.90) | | 0.78 (0.60, 1.03) | |
| **Hospital location/teaching status** | | | | |
| Urban teaching | ref | ref | ref | |
| Urban nonteaching | 1.63 (0.87, 3.04) | 1.67 (0.89, 3.12) | **1.31 (1.06, 1.63)** | |
| Rural | NA | NA | 1.03 (0.81, 1.32) | |
| **Year admission** | | | | |
| 2005-2010 | ref | | ref | ref |
| 2011-2014 | 0.83 (0.36, 1.88) | | 0.81 (0.60, 1.10) | 0.83 (0.61, 1.13) |
| 2015-2018 | 1.07 (0.51, 2.24) | | **0.57 (0.42, 0.76)** | **0.53 (0.39, 0.71)** |

[a] Excluded patients with in-hospital mortality. [b] LOS > 9 days. Significant values are shown in bold. LOS, length of stay; HMO, Health Maintenance Organization; CCI, Charlson Comorbidity Index; OR, odds ratio; aOR, adjusted OR. NA: not applicable, no event occurred in the category.

**Supplementary Table S2.** Associations between study variables and postoperative complications. (cont.).

| | Complications (any) | | Bleeding | | Pneumonia | |
|---|---|---|---|---|---|---|
| | **Univariable** | **Multivariable** | **Univariable** | **Multivariable** | **Univariable** | **Multivariable** |
| | **OR (95% CI)** | **aOR (95% CI)** | **OR (95% CI)** | **aOR (95% CI)** | **OR (95% CI)** | **aOR (95% CI)** |
| **Type of surgery** | | | | | | |
| Pure laparoscopic | ref | ref | ref | ref | ref | ref |
| Robot-assisted | 0.99 (0.78, 1.27) | **0.69 (0.54, 0.88)** | 1.17 (0.88, 1.57) | **0.73 (0.54, 0.99)** | **0.48 (0.27, 0.84)** | **0.49 (0.28, 0.86)** |
| **Age, years** | | | | | | |
| 50-59 | ref | ref | ref | ref | ref | |
| 60-69 | 1.10 (0.85, 1.41) | 1.02 (0.78, 1.33) | 1.03 (0.77, 1.38) | 1.00 (0.72, 1.39) | 1.15 (0.48, 2.76) | |
| 70-79 | 1.28 (1.00, 1.64) | 1.05 (0.79, 1.39) | 1.31 (0.99, 1.74) | 1.18 (0.84, 1.65) | 1.41 (0.61, 3.24) | |
| 80+ | **1.64 (1.23, 2.19)** | 1.23 (0.88, 1.72) | **1.51 (1.07, 2.13)** | 1.18 (0.79, 1.78) | 2.24 (0.93, 5.43) | |
| **Sex** | | | | | | |
| Male | 0.95 (0.79, 1.14) | | 0.84 (0.69, 1.03) | | 0.83 (0.48, 1.44) | |
| Female | ref | | ref | | ref | |
| **Race** | | | | | | |
| White | ref | | ref | | ref | ref |
| Black | 0.85 (0.60, 1.20) | | 0.85 (0.56, 1.27) | | 1.20 (0.44, 3.23) | 1.10 (0.41, 2.96) |
| Hispanic | 1.01 (0.68, 1.51) | | 0.87 (0.53, 1.42) | | 1.00 (0.24, 4.14) | 0.98 (0.23, 4.09) |
| Others | 0.68 (0.44, 1.06) | | 0.98 (0.62, 1.55) | | NA | NA |
| **Household income** | | | | | | |
| Quartile1 | 0.86 (0.68, 1.08) | | 0.93 (0.72, 1.20) | | 1.60 (0.83, 3.09) | |
| Quartile2 | 0.89 (0.72, 1.09) | | 0.87 (0.68, 1.12) | | 1.21 (0.62, 2.34) | |
| Quartile3 | 0.95 (0.78, 1.15) | | 0.96 (0.77, 1.20) | | 0.95 (0.48, 1.91) | |
| Quartile4 | ref | | ref | | ref | |
| **Insurance status** | | | | | | |
| Medicare/Medicaid | ref | ref | ref | ref | ref | ref |
| Private including HMO | **0.70 (0.59, 0.83)** | **0.76 (0.61, 0.93)** | **0.71 (0.58, 0.86)** | **0.77 (0.61, 0.97)** | **0.37 (0.19, 0.75)** | **0.50 (0.26, 0.96)** |
| Self-pay/no-charge/ other | 0.97 (0.64, 1.48) | 0.96 (0.63, 1.46) | 0.96 (0.61, 1.52) | 0.93 (0.58, 1.48) | 0.73 (0.23, 2.35) | 0.50 (0.07, 3.70) |
| **CCI** | | | | | | |
| 0-1 | NA | NA | NA | NA | NA | NA |
| 2-3 | ref | ref | ref | ref | ref | ref |
| 4-5 | **1.69 (1.42, 2.02)** | **1.82 (1.51, 2.20)** | **1.42 (1.17, 1.73)** | **1.53 (1.24, 1.90)** | **2.02 (1.21, 3.37)** | 1.40 (0.79, 2.48) |
| 6 | **1.40 (1.04, 1.86)** | **1.69 (1.25, 2.29)** | 1.18 (0.85, 1.65) | **1.53 (1.07, 2.18)** | **2.56 (1.25, 5.23)** | 1.97 (0.93, 4.19) |
| **Hospital bed size** | | | | | | |
| Large | ref | | ref | ref | ref | |
| Medium | 0.83 (0.68, 1.01) | | **0.74 (0.59, 0.94)** | 0.90 (0.71, 1.15) | 1.16 (0.67, 2.01) | |
| Small | 0.87 (0.70, 1.10) | | 0.91 (0.70, 1.19) | **0.77 (0.59, 0.99)** | 0.90 (0.45, 1.80) | |
| **Hospital location/teaching status** | | | | | | |
| Urban teaching | ref | ref | ref | ref | ref | |
| Urban nonteaching | **1.73 (1.41, 2.11)** | **1.42 (1.15, 1.75)** | **1.71 (1.33, 2.19)** | **1.33 (1.03, 1.72)** | 0.82 (0.43, 1.56) | |
| Rural | **1.14 (1.04, 1.26)** | **1.21 (1.09, 1.35)** | **1.37 (1.23, 1.54)** | **1.39 (1.19, 1.63)** | 1.15 (0.89, 1.47) | |
| **Year admission** | | | | | | |
| 2005-2010 | ref | ref | ref | ref | ref | |
| 2011-2014 | 0.93 (0.72, 1.19) | 0.94 (0.72, 1.22) | 0.83 (0.64, 1.08) | 0.83 (0.63, 1.09) | 0.57 (0.28, 1.13) | |
| 2015-2018 | **0.21 (0.16, 0.27)** | **0.20 (0.15, 0.26)** | **0.10 (0.07, 0.14)** | **0.09 (0.07, 0.13)** | 0.69 (0.37, 1.29) | |

Significant values are shown in bold. HMO, Health Maintenance Organization; CCI, Charlson Comorbidity Index; OR, odds ratio; aOR, adjusted OR. NA: not applicable, no event occurred in the category.

**Supplementary Table S3.** Associations between study variables and postoperative complications.

| | Infection | | Wound complications | | Sepsis | |
|---|---|---|---|---|---|---|
| | Univariable | Multivariable | Univariable | Multivariable | Univariable | Multivariable |
| | OR (95% CI) | aOR (95% CI) | OR (95% CI) | aOR (95% CI) | OR (95% CI) | aOR (95% CI) |
| **Type of surgery** | | | | | | |
| Pure laparoscopic | ref | ref | ref | ref | ref | ref |
| Robot-assisted | 0.81 (0.54, 1.22) | **0.55 (0.36, 0.85)** | **0.47 (0.30, 0.72)** | **0.33 (0.20, 0.54)** | **0.48 (0.34, 0.67)** | **0.49 (0.34, 0.69)** |
| **Age, years** | | | | | | |
| 50-59 | ref | | ref | | ref | ref |
| 60-69 | 1.34 (0.89, 2.03) | | **0.57 (0.34, 0.93)** | | 1.27 (0.80, 2.03) | 1.14 (0.69, 1.89) |
| 70-79 | 1.20 (0.78, 1.85) | | 0.87 (0.53, 1.42) | | **1.62 (1.03, 2.56)** | 1.34 (0.79, 2.27) |
| 80+ | **1.60 (1.01, 2.53)** | | 1.01 (0.56, 1.84) | | **2.12 (1.31, 3.45)** | 1.70 (0.97, 2.97) |
| **Sex** | | | | | | |
| Male | 1.08 (0.78, 1.47) | | 1.16 (0.73, 1.85) | | 1.04 (0.75, 1.43) | |
| Female | ref | | ref | | ref | |
| **Race** | | | | | | |
| White | ref | | ref | | ref | |
| Black | 1.01 (0.56, 1.81) | | 0.29 (0.07, 1.20) | | 0.89 (0.48, 1.67) | |
| Hispanic | 1.20 (0.63, 2.30) | | 1.02 (0.37, 2.82) | | 1.44 (0.78, 2.65) | |
| Others | 0.59 (0.29, 1.20) | | 0.66 (0.24, 1.80) | | 0.55 (0.24, 1.25) | |
| **Household income** | | | | | | |
| Quartile1 | ref | | ref | ref | ref | ref |
| Quartile2 | **0.62 (0.42, 0.93)** | | **0.50 (0.28, 0.90)** | **0.48 (0.27, 0.86)** | 0.66 (0.44, 1.00) | **0.64 (0.42, 0.96)** |
| Quartile3 | 0.77 (0.56, 1.07) | | 0.90 (0.57, 1.42) | 0.85 (0.54, 1.36) | 0.76 (0.54, 1.06) | **0.70 (0.50, 0.98)** |
| Quartile4 | 0.78 (0.57, 1.08) | | **0.55 (0.35, 0.87)** | **0.56 (0.36, 0.89)** | **0.62 (0.44, 0.87)** | **0.59 (0.42, 0.83)** |
| **Insurance status** | | | | | | |
| Medicare/Medicaid | ref | | ref | | ref | ref |
| Private including HMO | 0.79 (0.59, 1.05) | | 0.87 (0.60, 1.24) | | **0.66 (0.49, 0.89)** | 0.88 (0.62, 1.25) |
| Self-pay/ no-charge/ other | 1.01 (0.52, 1.94) | | 1.00 (0.41, 2.42) | | 1.25 (0.67, 2.35) | 1.48 (0.73, 3.00) |
| **CCI** | | | | | | |
| 0-1 | | | | | | |
| 2-3 | ref | ref | ref | ref | ref | ref |
| 4-5 | **1.86 (1.41, 2.46)** | **2.00 (1.50, 2.66)** | **1.85 (1.28, 2.68)** | **1.97 (1.35, 2.87)** | **2.13 (1.61, 2.82)** | **1.98 (1.48, 2.65)** |
| 6 | **1.92 (1.28, 2.88)** | **2.36 (1.54, 3.61)** | **1.15 (0.57, 2.32)** | **1.30 (0.63, 2.67)** | **1.67 (1.06, 2.65)** | 1.52 (0.95, 2.43) |
| **Hospital bed size** | | | | | | |
| Large | ref | ref | ref | | ref | |
| Medium | **0.58 (0.41, 0.83)** | **0.68 (0.47, 0.98)** | 0.94 (0.61, 1.43) | | 0.85 (0.62, 1.15) | |
| Small | **0.55 (0.34, 0.92)** | **0.50 (0.31, 0.83)** | 0.60 (0.34, 1.07) | | 0.63 (0.40, 1.00) | |
| **Hospital location/teaching status** | | | | | | |
| Urban teaching | ref | ref | ref | ref | ref | ref |
| Urban nonteaching | **1.49 (1.08, 2.06)** | 1.18 (0.85, 1.65) | 1.52 (0.92, 2.50) | 1.15 (0.68, 1.93) | **1.69 (1.26, 2.27)** | **1.65 (1.23, 2.22)** |
| Rural | 0.99 (0.85, 1.14) | 0.86 (0.72, 1.03) | **2.05 (1.67, 2.51)** | 1.35 (1.00, 1.82) | **1.97 (1.70, 2.30)** | **1.73 (1.40, 2.14)** |
| **Year admission** | | | | | | |
| 2005-2010 | ref | ref | ref | ref | ref | |
| 2011-2014 | 0.99 (0.70, 1.40) | 0.99 (0.70, 1.40) | 0.71 (0.44, 1.16) | 0.77 (0.47, 1.25) | 1.09 (0.69, 1.73) | |

| | | | | | |
|---|---|---|---|---|---|
| 2015-2018 | **0.19 (0.13, 0.28)** | **0.17 (0.11, 0.25)** | **0.22 (0.13, 0.38)** | **0.20 (0.12, 0.35)** | 0.87 (0.56, 1.37) |

Significant values are shown in bold. HMO, Health Maintenance Organization; CCI, Charlson Comorbidity Index; OR, odds ratio; aOR, adjusted OR.
